# Semi-quantitative weight-bearing assessment of knee osteoarthritis: COAKS (CT Osteoarthritis Knee Score) reliability

**DOI:** 10.1016/j.ostima.2024.100243

**Published:** 2024-07-31

**Authors:** Neil A. Segal, Zehra Akkaya, Justyn H Jeon, Tom Turmezei

**Affiliations:** a University of Kansas Medical Center, Kansas City, KS, USA; b The University of Iowa, Iowa City, IA, USA; c Ankara University Ibni Sina Hospital, Ankara, Turkey; d University of California, San Francisco, San Francisco, CA, USA; e Norfolk and Norwich University Hospital, Norwich, UK; f University of East Anglia, Norwich, UK

**Keywords:** Osteoarthritis, Weight-bearing, Computed tomography, Imaging, Score

## Abstract

**Objective:**

Our aims were to 1) introduce the semi-quantitative CT Osteoarthritis Knee Score (COAKS); and 2) report intra- and inter-observer reproducibility.

**Design:**

Weight-bearing CT (WBCT) images of 106 participants were reviewed to develop the COAKS system and create a standardized atlas. Images of 10 knees were used to train musculoskeletal radiologists with the atlas. Once trained, two radiologists independently scored 35 knees on two occasions using reformatted images in orthogonal planes. Joint space narrowing (JSN), osteophytes, subchondral cysts and subchondral sclerosis were scored (0–3 scale) in the medial tibiofemoral, lateral tibiofemoral, patellofemoral, and proximal tibiofibular compartments. Weighted kappa statistics were calculated for intra- and inter-observer reliability. Compartment feature scores were plotted as heat maps for each knee to illustrate OA severity and location.

**Results:**

Scoring for nearly all features in all compartments had substantial to near-perfect reliability (0.61–1.00). Both inter- and intra-observer results combined across all compartments demonstrated near-perfect agreement for JSN (0.87 and 0.86) and subchondral cysts (0.84 for both) and substantial agreement for osteophytes (0.79 and 0.74) and subchondral sclerosis (0.66 and 0.67).

**Conclusions:**

COAKS is a feasible, multiplanar, semi-quantitative, compartment-by-compartment WBCT-based knee OA scoring system that demonstrates substantial to near-perfect intra- and inter-observer reliability. The capacity of COAKS to characterize the location and severity of OA in the weight-bearing knee could enable patient stratification, selection, and longitudinal monitoring of structural disease severity in clinical trials and cohort studies.

**Abbreviations**
CT: computed tomography
COAKS: computed tomography osteoarthritis knee score
FF-XR: fixed-flexion radiography
JSN: joint space narrowing
JSW: joint space width
LTF: lateral tibiofemoral
MRI: magnetic resonance imaging
MOAKS: magnetic resonance imaging osteoarthritis Knee Score
MTF: medial tibiofemoral
OA: osteoarthritis
PF: patellofemoral
PTF: proximal tibiofibular
WBCT: weight bearing computed tomography

## 1 Introduction

Structural changes in osteoarthritis (OA) of the knee are often evaluated with semi-quantitative scoring systems based on imaging. For bony changes, 2-dimensional (2-D) standing fixed-flexion radiography (FF-XR) is frequently used for knee OA diagnosis and longitudinal assessment of joint space narrowing (JSN) in FDA-approved clinical trials [1]. However, joint positioning and beam angle variability can pose challenges in radiographic evaluation due to the 2-D representation of 3-D structures obscuring anatomy [2,3], which limits sensitivity and responsiveness to structural change [4]. Recently, weight-bearing computed tomography (WBCT) has been introduced, providing several advantages. By offering 3-D weight-bearing imaging in which menisci and cartilage are compressed and muscle forces are applied, the menisci, cartilage, ligaments, and knee joint space width (JSW) can be assessed in a functional configuration and without bony overlap.

WBCT has been found to have higher sensitivity, accuracy, and predictive value for detecting osteophytes and subchondral cysts than FF-XR [5]. 3-D JSW on WBCT correlates better with cartilage damage than 2-D JSW on FF-XR [6,7], and the responsiveness of 3-D JSW on WBCT for change over 24 months (SRM: −1.75 to −2.88) is substantially better than JSWx on FF-XR (−0.11 to −0.14) [4]. Also, when using articular cartilage morphology on MRI as the reference standard, tibiofemoral 3-D JSW on WBCT correlates more highly (*r* = 0.84) than minimal JSW on FF-XR (*r* = 0.66) [6], and sensitivity and accuracy for patellofemoral cartilage lesions are higher on WBCT than on FF-XR [8]. Thus, WBCT imaging biomarkers have the potential to stratify patients through quickly and inexpensively characterizing OA features not evident on radiographs.

Cardinal radiographic features of knee OA include bony features (osteophytes, subchondral cysts and sclerosis) and joint space narrowing. Weight-bearing is essential for useful measurement of JSN and CT is the gold standard for visualizing bony features. Semi-quantitative scoring techniques have been instrumental in characterizing knee OA severity [9] and in demonstrating hip and whole-body OA load [[9], [10], [11]].

Considering the increasing availability and advantages of WBCT over radiographs, a semi-quantitative scoring system could offer improved longitudinal evaluation and characterization of knee OA structural severity and location. Such a system would work by adopting and fusing elements of radiographic feature assessment (such used in OARSI grading) [12] with an approach to 3-D imaging dataset evaluation (such as taken by the MRI Osteoarthritis Knee Score, MOAKS [9] or ROAMES [13]). Such a fused approach would take the best of both modalities by offering a 3-D evaluation of the weight bearing knee joint with superior visualization of mineralized tissues. Given prior work establishing the diagnostic value, reproducibility, and validity of measures of knee OA on WBCT, and the acquisition of WBCT in large cohort and interventional studies, we developed the CT OA Knee Score (COAKS). The purposes of this report are to introduce the COAKS instrument and to report its inter- and intra-observer reliability.

## 2 Methods

COAKS was developed using WBCT images acquired with isotropic resolutions between 250 and 300 µm on two WBCT models (OnSight, Carestream Health, Inc., Rochester, NY; and LineUp, Curvebeam, Hatfield, PA), with an effective dose of 0.1mSv, equivalent to the average environmental radiation experienced by a person living at sea level for one week. This study was performed in accordance with the provisions of the Declaration of Helsinki, and image acquisition was approved by the University of Kansas Institutional Review Board (STUDY00142926 and STUDY00145012). All participants participated in an IRB-approved consent process, culminating in written informed consent given prior to inclusion.

In total, WBCT images were acquired for 106 knee pairs in an approximately 20° fixed flexion standing position. The study imaging data sets underwent a preliminary multiplanar review by a consultant musculoskeletal radiologist with 14 years of experience in musculoskeletal radiology (TT), to select the side for study inclusion, excluding sides with evidence of intervention or incomplete coverage of the knee joint. After developing the scoring system with TT, all WBCT images were initially graded by JHJ, a musculoskeletal radiology fellow with six years of clinical radiology experience, two in musculoskeletal imaging. Atlas examples were selected from this initial grading after review and confirmation of feature score by TT, who selected 10 test scans for training and 35 study scans for atlas verification with sight-only of JHJs initial scoring. Training cases were selected by TT to provide a range of overall joint disease severity with two cases having no or little evidence of osteoarthritis, three with advanced disease, and five in between these extremes. Study cases were selected according to their score profile in order to include at least one of each initial score in each category. The single selected knee was evaluated in each set, pre-defined in the electronic scoresheet (below). The test set was blindly evaluated by two musculoskeletal radiologists with 14 and 10 years of experience respectively (TT and ZA) in two separate sittings on different viewing platforms, coming together to review performance after each sitting and adjusting feature scores or verbal definitions by consensus.

Thirty-five study scans were blindly and independently evaluated by TT and ZA without any communication during this study period. ZA repeated the scoring after a 4-month washout period, with cases re-ordered and IDs re-blinded. While MRI semi-quantitative scoring systems commonly subdivide the joint into 14 articular sub-regions, through iterative development and refinement, on the advice of 6 experienced radiologists the COAKS instrument was streamlined to focus on the four pertinent compartments: medial tibiofemoral (MTF), lateral tibiofemoral (LTF), patellofemoral (PF), and proximal tibiofibular (PTF). Four features were scored 0–3, analogous to the OARSI and MOAKS scoring systems [9,12], in each of four compartments in any of the three orthogonal imaging planes pre-defined in the atlas. The boundary between the patellofemoral and tibiofemoral compartments is the same as that for MOAKS, with the rest of the compartments logically discernable from this [9].

### 2.1 COAKS assessment

The COAKS guide and atlas (see supplementary material) is intended to be used for scoring WBCT of the knee using any available DICOM image viewing software with 3-D multiplanar reformat capability. Recommended viewer settings include a screen zoom of 300 %, Window Level (WL)=575, and Window Width (WW)=2325. Readers set up orthogonal review planes as follows:

• Coronal plane: navigate to the central aspect of the MTF compartment.
• Sagittal plane: align the axial plane to the anterior and posterior margins of the tibial plateau.
• Axial plane: set the coronal plane to a line tangential to the posterior aspect of the posterior femoral condyles.

**Fig. 1:**
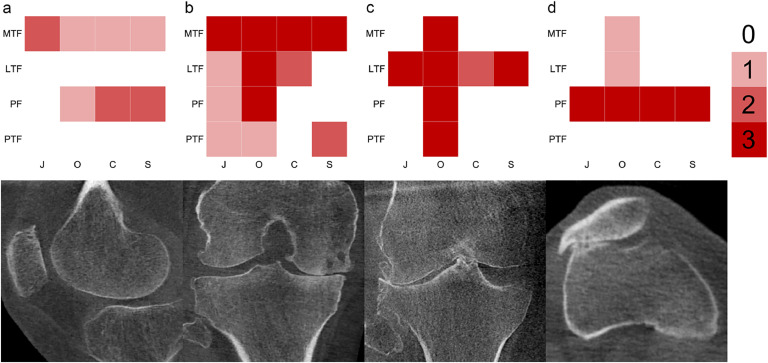
Heatmaps selected from the study cohort to demonstrate different patterns of structural disease as assessed from WBCT using the COAKS construct. Selected WBCT images are also representative of the three orthogonal imaging review planes. Color saturation increasing from white to red in three steps represents the scoring of 0–3 (see key); note that not all features in the heat maps will be seen in a single image capture because scores are assessed using multiplanar reformats for the whole compartment. **a** MTF + PF involvement (sagittal). **b** Advanced MTF involvement with multicompartment features (coronal). **c** Advanced LTF phenotype with severe multicompartmental osteophytosis but no other features (coronal). **d** PF involvement with small tibiofemoral osteophytes only (transverse). Key: *J* = JSN; *O* = osteophytes; C = subchondral cysts; *S* = subchondral sclerosis. MTF = medial tibiofemoral; LTF = lateral tibiofemoral; PF = patellofemoral; PTF = proximal tibiofibular.

Readers may navigate across and between compartments in any of the pre-selected planes, but without changing orientation in order to provide a compartment-by-compartment score profile. Examples of the three imaging review planes are shown in Fig. 1 *bottom*.

### 2.2 COAKS atlas construction

**Fig. 2:**
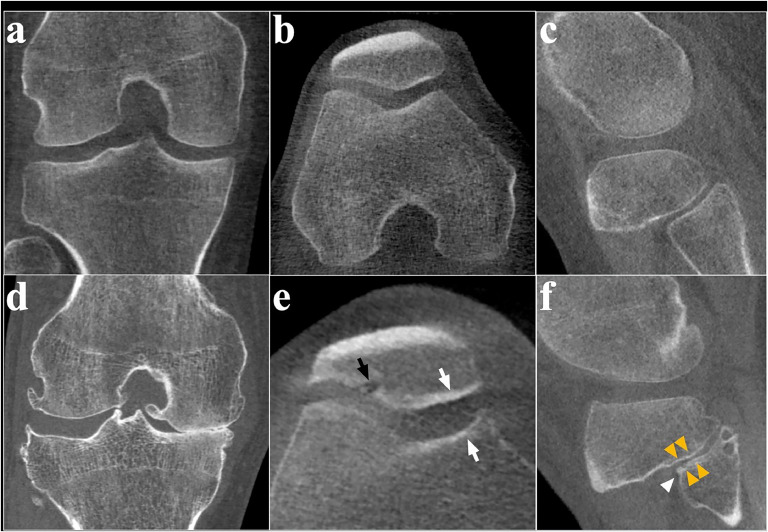
*(Top Row)* Coronal (a), axial (b) and sagittal oblique (c) views representing examples of normal-appearing WBCT images of the medial and lateral tibiofemoral, patellofemoral and proximal tibiofibular joints respectively: all compartmental feature scores would be zero, presuming no other features were present in the rest of the imaging. *(Bottom row)* Coronal reformatted WBCT image (d) representing joint space narrowing of the medial tibiofemoral compartment with osteophytes more extensive in the lateral TF compartment (but still a score of 3 in the medial and lateral TF compartments); Axial view (e) demonstrating a large subchondral cyst *(black arrow)* on the lateral patellar facet and medial patellar and trochlear subchondral sclerosis *(white arrows);* and a sagittal oblique reformatted WBCT image (f) demonstrating a tiny osteophyte of the proximal tibiofibular joint *(single arrow head)* and joint space narrowing and mild subchondral sclerosis anteriorly *(double arrow heads).*

Example images were provided for all compartments. The following features were scored from across each of the compartments using any of the preset planes with the most severe example from across the compartment taken as the final feature score (Fig. 2).

• Joint space Narrowing: “Narrowing of the distance between opposing subchondral bone articular surfaces from any cause”— none (0), <50 % (1), >50 % (2), complete or near-complete (3).
• Osteophytes: “A bony outgrowth or spur arising at the junction between articular cartilage and bone (at a marginal location) excluding enthesophytes at the point of ligament or tendon insertion and lesions within cartilage.”[9,12]— None (0); small (1); medium (2); large (3).
• Subchondral cysts: “A low-density region in subchondral bone interrupting the normal bony architecture, with or without a sclerotic rim. A connection with the articular surface need not be visible.”[11]— None (0); one cyst at a single location (1); multiple cysts on the same side of the joint (2); cysts (single or multiple) on both sides of the joint (3). NB: cysts need only be in the same compartment and not necessarily just the same review image to result in a score of 2 or 3.
• Subchondral sclerosis: “Subchondral bone plate thickening or increased subchondral trabecular bone density, beyond normal in load bearing or contact regions.”— None (0); possible or suspected (1); definite (but not grade 3) (2); severe with bony eburnation, collapse, erosion, or deformity (3)

The atlas includes a full descriptive and visual guide, with instructions for setting up for review, scoring each feature in each compartment, and examples of potential pitfalls (e.g., misidentifying intra-osseous cysts extending through the cruciate or meniscal insertions as a subchondral cyst).

Cloud-shared score sheets (Google Sheets; Google LLC, CA, USA) with the case ID, side, and all feature score definitions permitted score selection for each compartment/feature from a drop-down menu and text entry to comment. Blinded to each other's performance, both readers scored each feature in each compartment. Scores were automatically summed for each feature, for each compartment, and for the whole joint.

### 2.3 Statistical methods

Inter- (TT vs ZA) and intra-rater (ZA) agreement for each COAKS feature was calculated for each compartment and across the whole knee joint using weighted Kappa (with 95 % confidence intervals) [14] in a custom MATLAB (R2023a, The MathWorks, Inc., MA, USA) script. Strength of agreement based on kappa coefficients was interpreted as: ≤0 = poor, 0.01–.20 = slight, 0.21–.40 = fair, 0.41–.60 = moderate, 0.61–.80 = substantial, and 0.81–1 = almost perfect[15]. Heatmaps indicating location and severity of OA features were generated from a custom MATLAB script for each knee, displaying the score for each feature in each compartment in a red color-shaded four-by-four grid.

## 3 Results

On average, it took less than 5 min to score a single knee. Inter- and intra-observer weighted Kappa scores for each compartment and each feature are presented in Table 1, along with summary weighted Kappa scores for each feature across all compartments.

**Table 1:** Inter- and intra-observer weighted Kappa (**κ**_w_) and 95 % confidence interval (CI) for COAKS features in each compartment.

		Inter-observer Reliability			Intra-observer Reliability		
		**κ**_w_	95 % CI	Agreement	**κ**_w_	95 % CI	Agreement
**Medial tibiofemoral**	JSN	0.84	(0.80–0.88)	Near perfect	0.87	(0.85–0.89)	Near perfect
	Osteophytes	0.84	(0.79–0.89)	Near perfect	0.80	(0.74–0.86)	Substantial
	Sclerosis	0.69	(0.62–0.75)	Substantial	0.61	(0.55–0.68)	Substantial
	Cysts	0.91	(0.89–0.93)	Near perfect	0.80	(0.75–0.84)	Substantial
**Lateral tibiofemoral**	JSN	0.87	(0.84–0.90)	Near perfect	0.89	(0.86–0.91)	Near perfect
	Osteophytes	0.64	(0.54–0.73)	Substantial	0.67	(0.58–0.76)	Substantial
	Sclerosis	0.66	(0.58–0.74)	Substantial	0.68	(0.61–0.75)	Substantial
	Cysts	0.91	(0.89–0.93)	Near perfect	0.89	(0.87–0.91)	Near perfect
**Patellofemoral**	JSN	0.89	(0.86–0.91)	Near perfect	0.88	(0.86–0.91)	Near perfect
	Osteophytes	0.75	(0.68–0.81)	Substantial	0.68	(0.61–0.75)	Substantial
	Sclerosis	0.50	(0.41–0.58)	Moderate	0.50	(0.46–0.54)	Moderate
	Cysts	0.74	(0.69–0.78)	Substantial	0.88	(0.86–0.90)	Near perfect
**Proximal tibiofibular**	JSN	0.83	(0.80–0.85)	Near perfect	0.66	(0.58–0.74)	Substantial
	Osteophytes	0.85	(0.82–0.88)	Near perfect	0.62	(0.56–0.68)	Substantial
	Sclerosis	0.65	(0.60–0.70)	Substantial	0.52	(0.47–0.58)	Moderate
	Cysts	0.63	(0.59–0.68)	Substantial	0.58	(0.54–0.62)	Moderate
**Combined**	JSN	0.87	(0.86–0.89)	Near perfect	0.86	(0.83–0.88)	Near perfect
	Osteophytes	0.79	(0.76–0.82)	Substantial	0.74	(0.70–0.78)	Substantial
	Sclerosis	0.66	(0.63–0.70)	Substantial	0.67	(0.63–0.70)	Substantial
	Cysts	0.84	(0.82–0.86)	Near perfect	0.84	(0.82–0.86)	Near perfect

Other than subchondral sclerosis at the patellofemoral compartment (for inter- and intra-observer rating) and subchondral sclerosis and cysts at the PTF joint (intra-observer rating only) all results demonstrated at least substantial to near perfect reliability (in the 0.61–1.00 range). Both inter- and intra-observer results combined across all compartments demonstrated near-perfect agreement for JSN (0.87 and 0.86) and subchondral cysts (0.84 for both) and substantial agreement for osteophytes (0.79 and 0.74) and subchondral sclerosis (0.66 and 0.67).

Example heat maps for four knees with different patterns of OA features are shown in Fig. 1.

## 4 Discussion

COAKS offers a compartment-by-compartment WBCT-based semi-quantitative scoring system for mineralized structural disease features of knee OA that appears to have very good to excellent inter- and intra-rater reliability. Radiographs have long been considered the standard for OA structural disease assessment. The capability of WBCT to visualize joints in 3-D. however, offers the opportunity to set a new standard beyond radiography for characterization of OA severity and compartmental involvement, stratification and monitoring with respect to mineralized joint tissues. Compared to MRI, CT imaging is more efficient, cost-effective, and accessible. WBCT is increasingly utilized to acquire weight bearing knee imaging [5,16] at the same relative radiation level [17] as radiographs, making it applicable for large research studies and translation to the clinic.

The COAKS atlas provides the first whole joint CT-based scoring system of structural features in knee OA that also encompasses JSN from weight bearing acquisition. This type of scoring system has been available for knee radiographs [12] and MRI [9], and for CT of the hip [11] and whole body [10]. COAKS uses a hybrid approach to identify classical radiographic features of OA as described by Kellgren & Lawrence and OARSI grading [12,18], but is unique in including scoring of the PTF joint. The overlapping zonal assessment between COAKS and MRI-based MOAKS scoring [9] could enable cross-modality comparison, with MRI as part of a multimodal whole joint assessment and taking advantage of the growing application of WBCT to assess the joint space in 3-D.

The COAKS system was designed in consultation with a panel of musculoskeletal radiologists with substantial experience in OA imaging with the goal of delivering efficient, reliable, and reproducible scoring, while retaining the sensitivity to detect meaningful differences in compartmental involvement and severity between individuals and longitudinally within individuals. Alternatively, subregions can be collapsed to deliver a score by compartment (out of 12), feature (out of 12), or composite whole knee (out of 48, including the MTF, LTF, PF and PTF joints). The clinical utility of such derived scores needs to be established through additional research. Furthermore, heatmaps, such as the examples provided in Fig. 1 could enable convenient patient stratification or selection for inclusion in clinical trials.

Importantly, while responsible for lateral knee joint stability, load transmission, biomechanics, and knee pain, the PTF joint has not been included in other scoring systems. Damage to the PTF joint has been implicated in lateral knee pain[19]. Thus, it is significant that COAKS is the first scoring system to include this joint. With the increasing use of WBCT, assessment of this joint may provide novel insights into knee OA phenotypes and pain generators.

Limitations of this study include use of some WBCT images with image quality inferior to that of current WBCT. The scanners used in this study were more limited in anode power (mA), leading to photon starvation artefacts, particularly in obese patients. Current WBCT, however, has overcome this limitation through the introduction of x-ray parameters that improve soft tissue contrast, offering higher quality images over a range of body types and OA features. Thus, in addition to improving visualization of the features included in COAKS, scoring of the degree of meniscal extrusion on WBCT could be added in future work, improving characterization of early knee OA features. Another limitation was the presence of only two raters, although both are experienced musculoskeletal radiologists. External validation of COAKS by a broader range of scorers would contribute generalizable information on usability.

In conclusion, COAKS is a new system for multiplanar and multicompartmental evaluation of structural knee OA. This has been presented with an expert guide and atlas to support scoring, demonstrating substantial to near-perfect preliminary interobserver results. These data support that COAKS is robust with respect to scanner, imaging review platform, scan artefacts, and range of disease. Semi-quantitative scoring of OA can be presented by compartment, feature, or the whole joint, while 3-D structural classification is available through heat map visualization. As WBCT technology continues to develop and become more accessible, the COAKS system has the potential to improve patient stratification and selection for clinical trials beyond radiography and alongside MRI, while further validation could expedite clinical use.

## Funding

This research did not receive any specific grant from funding agencies in the public, commercial, or not-for-profit sectors.

## Declaration of competing interest

None.

## Appendix Supplementary materials

Image, application 1
