# Supplementary material for: Semi-quantitative weight-bearing assessment of knee osteoarthritis: COAKS (CT Osteoarthritis Knee Score) reliability
Source: Osteoarthr Imaging. 2024 Jul 31;4(3):100243. doi: 10.1016/j.ostima.2024.100243 (PMC13228664; doi:10.1016/j.ostima.2024.100243)
Supplement: Supplementary file 1 [file mmc1.pdf]

CT OSTEOARTHRITIS KNEE SCORE (COAKS) REVIEW GUIDE & ATLAS (v3)

SETUP

Use any DICOM image viewing software with 3-D multiplanar reformat capability. In Norwich we have used Horos.

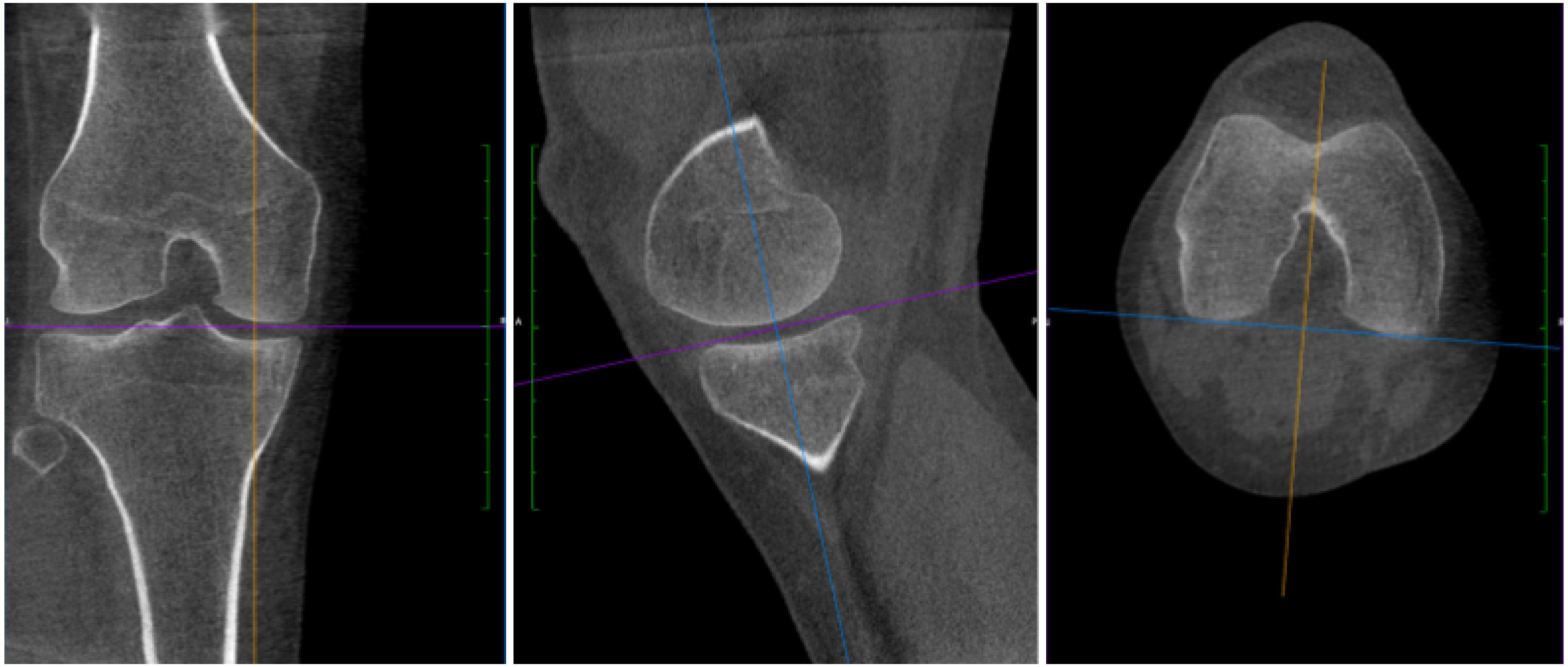

> In the coronal window (blue), navigate to the central aspect of the medial tibiofemoral compartment
> In the sagittal window (orange), align the axial plane (purple) to the anterior and posterior margins of the tibial plateau.
> In the axial window (purple), set the coronal plane (blue) to a line at a tangent to the posterior aspect of the posterior femoral condyles.
> Screen zoom to 300%.
> Window Level (WL) = 575 | Window Width (WW) = 2325 (In Norwich, this is preset COAKS, keyboard shortcut 1)

NORMAL COMPARTMENT EXAMPLES (ALL IMAGES RIGHT KNEE THROUGHOUT)

<table>
<tr><td>Medial tibiofemoral (coronal)</td><td>Lateral tibiofemoral (coronal)</td><td>Patellofemoral (axial)</td><td>Proximal tibiofibular (axial)</td></tr>
<tr><td>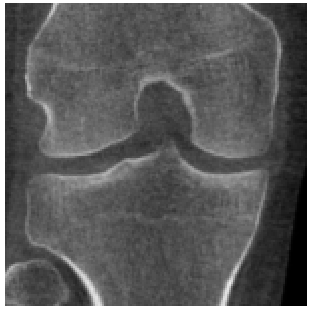</td><td>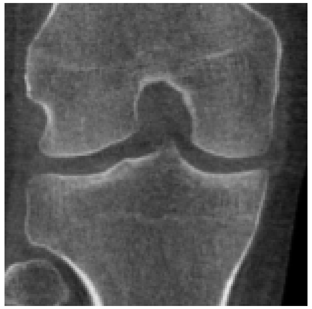</td><td>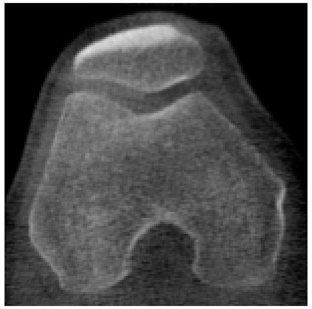</td><td>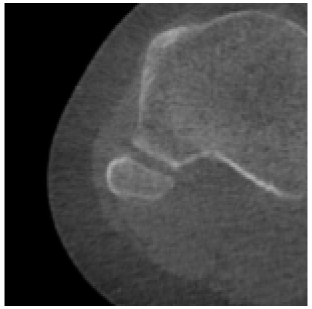</td></tr>
<tr><td>Medial tibiofemoral (sagittal)</td><td>Lateral tibiofemoral (sagittal)</td><td>Patellofemoral (sagittal)</td><td>Proximal tibiofibular (sagittal)</td></tr>
<tr><td>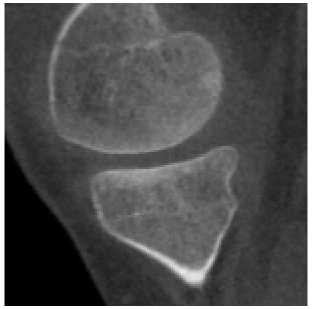</td><td>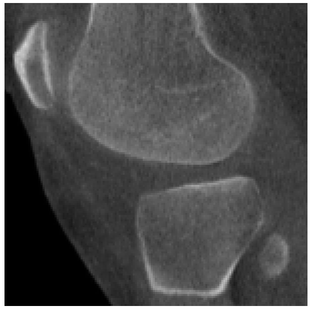</td><td>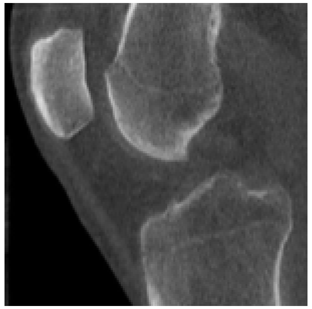</td><td>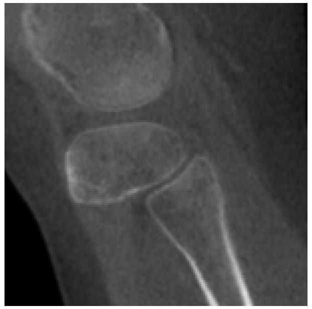</td></tr>
<tr><td>PF-TF boundary (sagittal)</td><td></td><td></td><td>Proximal tibiofibular (coronal)</td></tr>
<tr><td>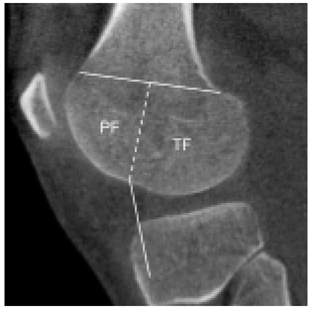</td><td colspan="2">The boundary between the patellofemoral (PF) and tibiofemoral (TF) compartments is defined in the sagittal plane as:

"the (dashed) line drawn from the centre point of the line across the junction between the femoral condyles and shaft to the line drawn tangentially up from the anterior margin of the tibial plateau articular surface where it meets the femoral subchondral bone."

This example is from the lateral compartment.</td><td>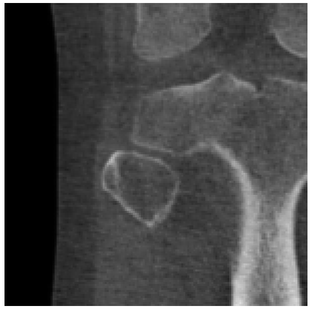</td></tr>
</table>

CONCEPT

The goal of COAKS is to create a new grading system for the mineralised structural features of osteoarthritis (OA).

The system has been designed specifically with weight bearing computed tomography (WBCT) in mind, since this allows evaluation of the weight bearing joint, which has become increasingly popular as a research and clinical investigation tool.

It is also compatible with non-weight bearing CT, but either joint space width is ignored, or it reserved only for comparison against other non-weight bearing cases; the same caveat applies to keeping the same weight bearing conditions within any project being scored.

The grading has been devised to score disease features help in the challenge of identifying onset and disease progression, but it can equally be used to stratify individuals by defining a CT heat map according to feature relationships and distribution in the four joint compartments at the knee: medial tibiofemoral (mTF), lateral tibiofemoral (lTF), patellofemoral (PF), and proximal tibiofibular (pTF).

Using a semi-quantitative compartment-by-compartment approach with CT similar to MOAKS may allow cross-modality comparison with MRI while also serving the growing use of WBCT. All cases in this guide are from cone beam WBCT.

In designing the COAKS system, we considered what should be easiest to score efficiently and reliably, while also aiming for sensitivity.

Having set up (as above), the reader is free to navigate using multiplanar reformats to assess each compartment according to the guide (below) for each feature, which not only includes the grade categories for each feature but also the recommended planes to use.

Four features are scored from 0 to 3 for each compartment: joint space narrowing (J), osteophytes (O), subchondral cysts (C), and subchondral sclerosis (S). The WORST example is taken as the final feature score for that given compartment.

All examples in this guide are shown as a weight bearing RIGHT knee from cone beam CT acquisitions. A single plane showing the graded feature is given as a representative example in each case, noting that this means no more severe example of that features is then presumed to be present outside of the given imaging review plane (in reality, this will often not be the case).

At the end of the guide we also include common pitfalls and exclusions.

NB: We have populated our guide prior to this testing, but there is an opportunity after the reproducibility exercise for additional consensus reading to deliver what we aim to be a definitive CT grading system for knee osteoarthritis akin to the OARSI grading atlas (https://www.oarsijournal.com/article/S1063-4584(06)00328-1/fulltext).

JOINT SPACE NARROWING

Definition

"Narrowing of the distance between opposing subchondral bone articular surfaces from any cause"

Notes:

- Recommended planes for assessment are:
  - coronal and sagittal for tibiofemoral compartments;
  - axial for the patellofemoral compartment;
  - axial, sagittal, and coronal for the proximal tibiofibular compartment.
- Marginal or central osteophytes (including tibial spine spiking) do not count towards joint space narrowing.
- Narrowing in either side (medial and/or lateral) counts for the patellofemoral compartment.

JOINT SPACE NARROWING GRADE 0 = none			
Medial tibiofemoral (coronal)	Lateral tibiofemoral (coronal)	Patellofemoral (axial)	Proximal tibiofibular (axial)
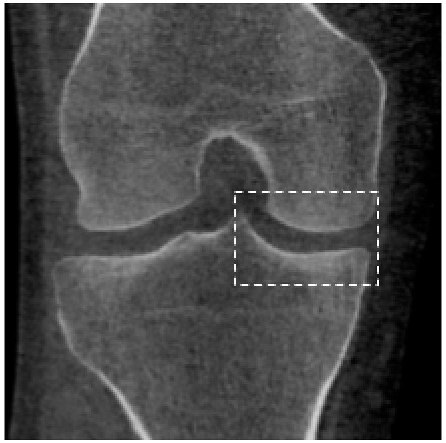	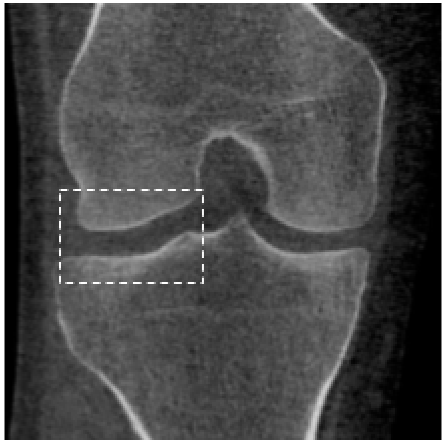	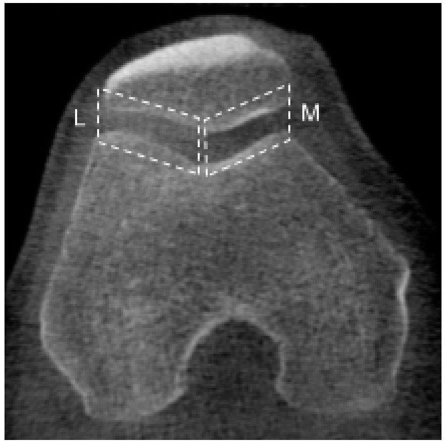	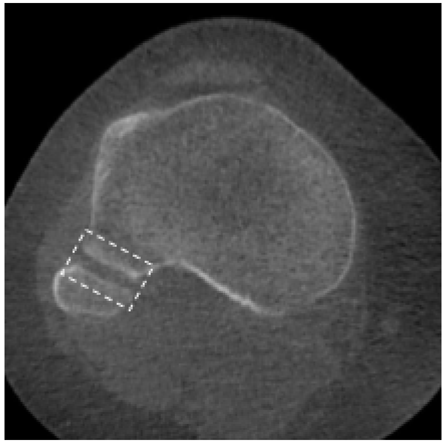

JOINT SPACE NARROWING GRADE 1 = up to 50% loss of joint space			
Medial tibiofemoral (coronal)	Lateral tibiofemoral (coronal)	Patellofemoral (axial)	Proximal tibiofibular (axial)
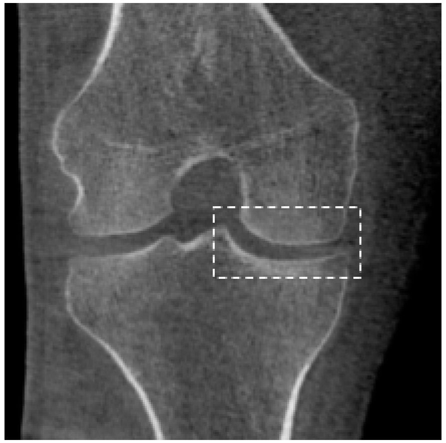	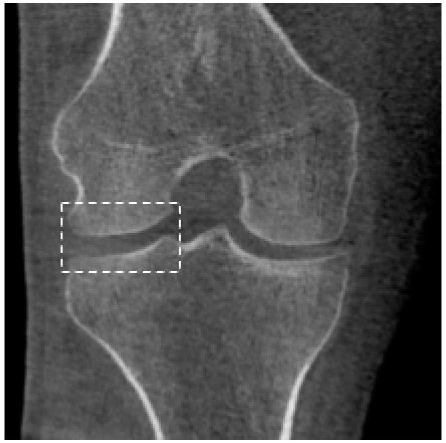	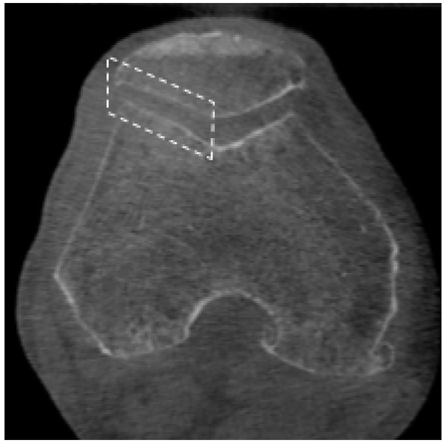	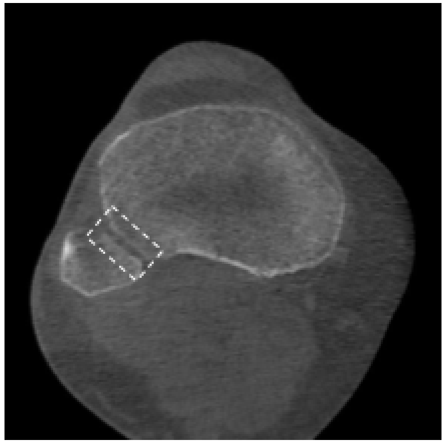

JOINT SPACE NARROWING GRADE 2 = >50% loss of joint space but not bone-on-bone, and no articular surface deformity			
Medial tibiofemoral (coronal)	Lateral tibiofemoral (coronal)	Patellofemoral (axial)	Proximal tibiofibular (axial)
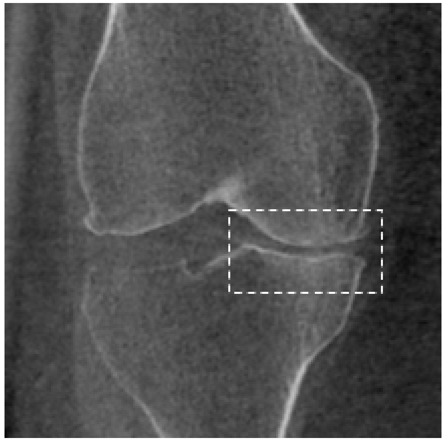	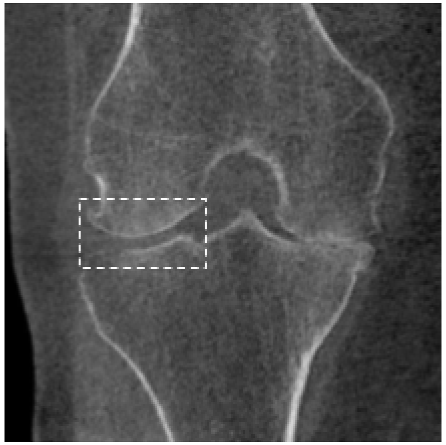	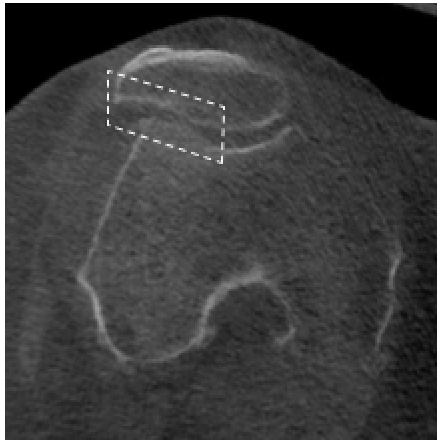	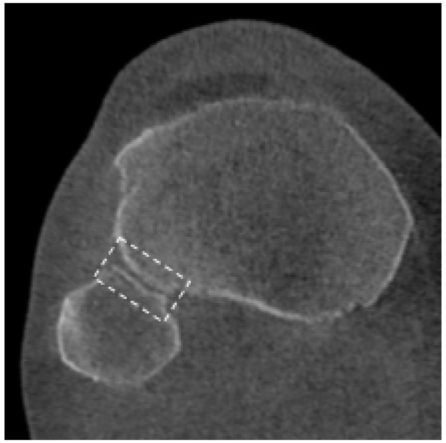

JOINT SPACE NARROWING GRADE 3 = Bone-on-bone, ankylosis, or near-complete joint space loss with articular surface deformity			
Medial tibiofemoral (coronal)	Lateral tibiofemoral (coronal)	Patellofemoral (axial)	Proximal tibiofibular (axial)
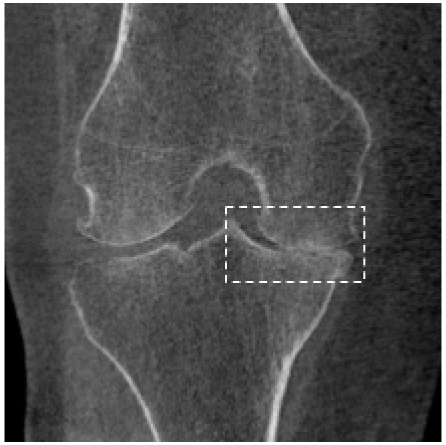	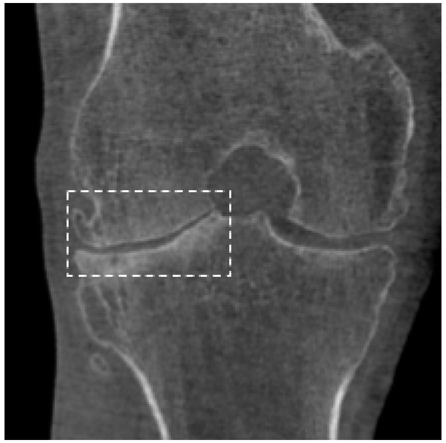	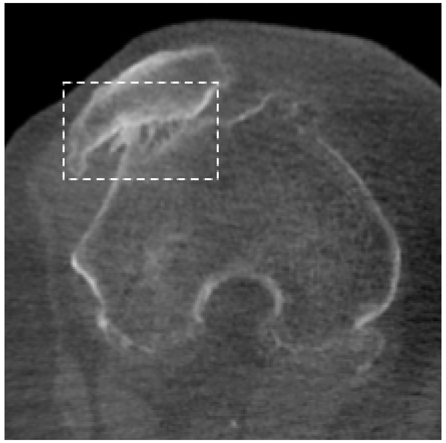	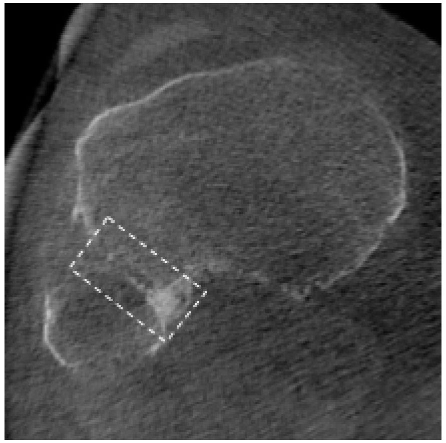

OSTEOPHYTES

Definition:

"A bony outgrowth or spur arising at the junction between articular cartilage and bone (at a marginal location) excluding enthesophytes at the point of ligament or tendon insertion and lesions within cartilage (i.e. central)."

Notes:

- Recommended planes for assessment are:
  - coronal and sagittal for tibiofemoral compartments;
  - axial and sagittal for the patellofemoral compartment;
  - axial, sagittal, and coronal for the proximal tibiofibular compartment.
- Score the largest osteophyte in the compartment as extent of protuberance out from the bone surface, not along the bone margin of the joint.
- Central osteophytes (i.e. at the articular surface) are not included in the assessment.
- Ignore detached osteophytes that can be difficult to distinguish from intra-articular bodies.
- Osteophytes at either of the tibial spines are scored in the respective medial or lateral tibiofemoral compartment.
- Osteophytes at the intercondylar notch of the femur are scored in the respective medial or lateral tibiofemoral compartment.
- On the right is a reminder of the small-medium-large (1-2-3) size breakdown in MOAKS.

996 D.J. Hunter et al. / Osteoarthritis and Cartilage 19 (2011) 990–1002

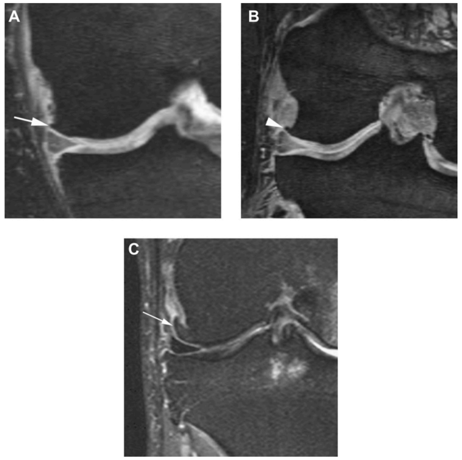

Fig. 7. Scoring of osteophytes. Grade 0 = none, grade 1 = small, grade 2 = medium and grade 3 = large. A. Grade 1 osteophyte medial femur. B. Grade 2 osteophyte lateral femur. C. Grade 3 osteophyte lateral femur. Size of osteophyte should reflect protuberance (how far the osteophyte extends from the joint) rather than total volume of osteophyte.

OSTEOPHYTE GRADE 0 = None			
Medial tibiofemoral (coronal)	Lateral tibiofemoral (coronal)	Patellofemoral (axial)	Proximal tibiofibular (axial)
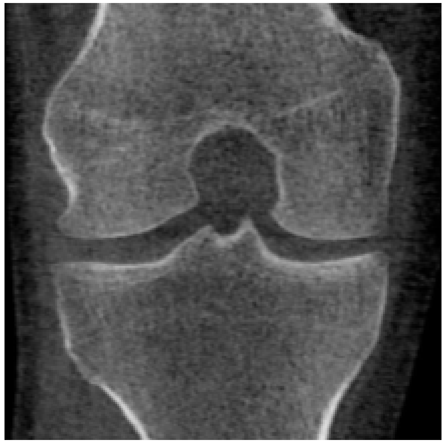	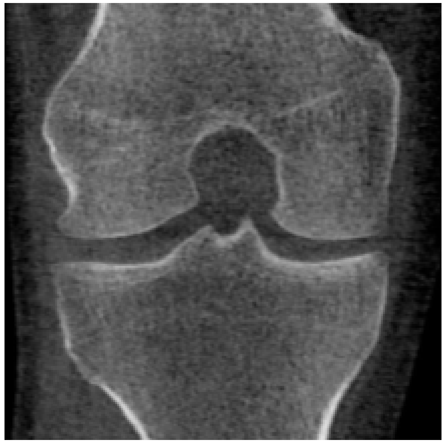	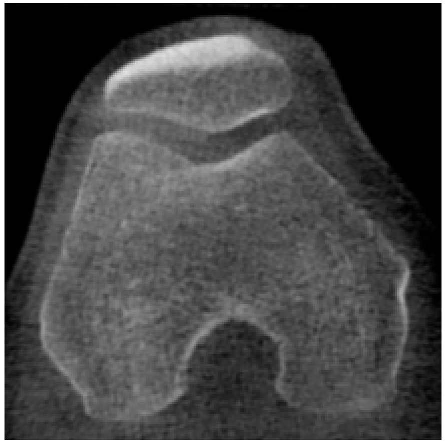	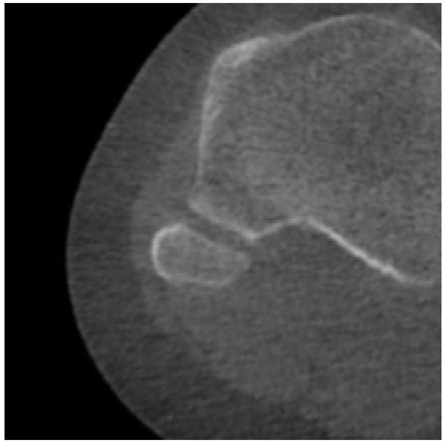

OSTEOPHYTE GRADE 1 = Small			
Medial tibiofemoral (coronal)	Lateral tibiofemoral (coronal)	Patellofemoral (axial)	Proximal tibiofibular (coronal)
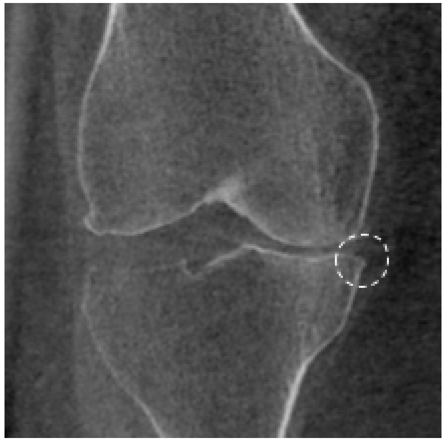	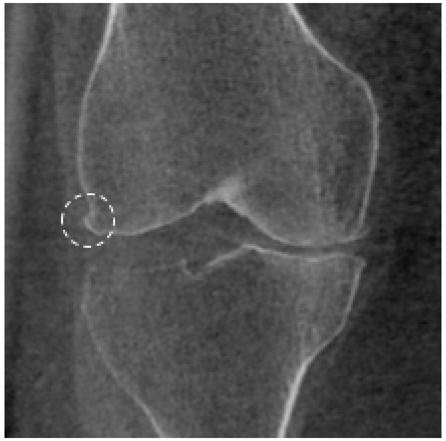	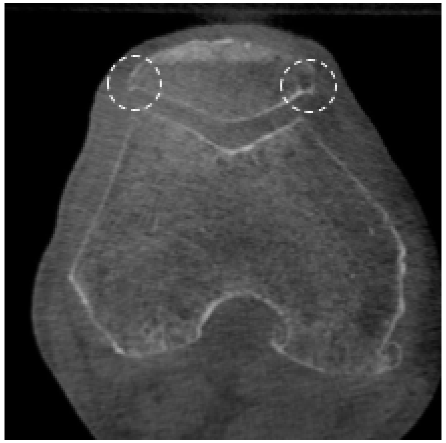	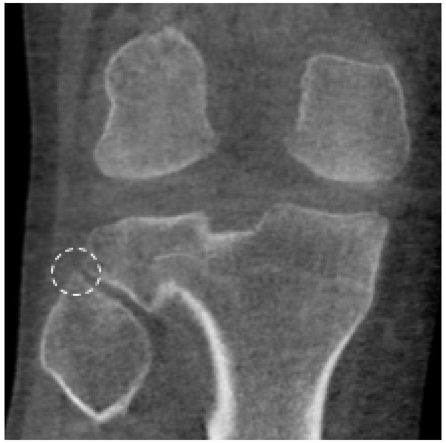

OSTEOPHYTE GRADE 2 = Medium			
Medial tibiofemoral (coronal)	Lateral tibiofemoral (coronal)	Patellofemoral (axial)	Proximal tibiofibular (coronal)
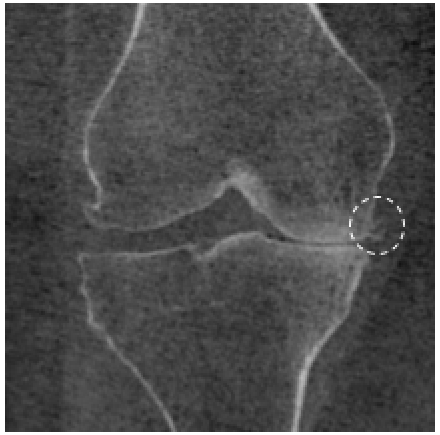	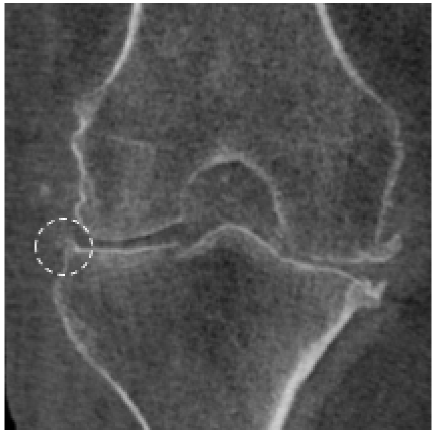	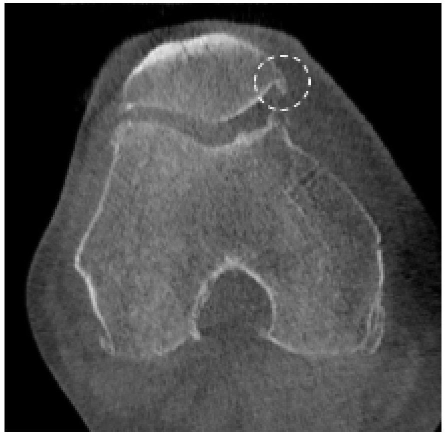	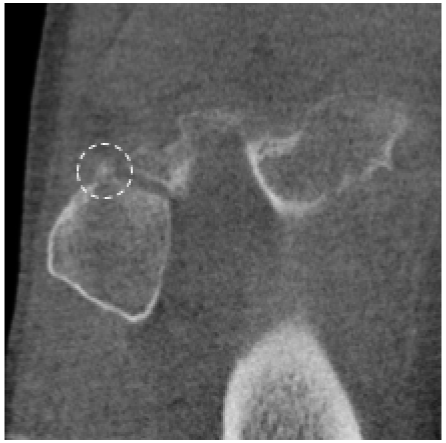

OSTEOPHYTE GRADE 3 = Large			
Medial tibiofemoral (coronal)	Lateral tibiofemoral (coronal)	Patellofemoral (axial)	Proximal tibiofibular (axial)
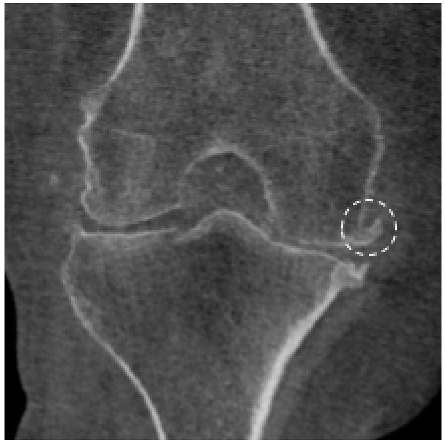	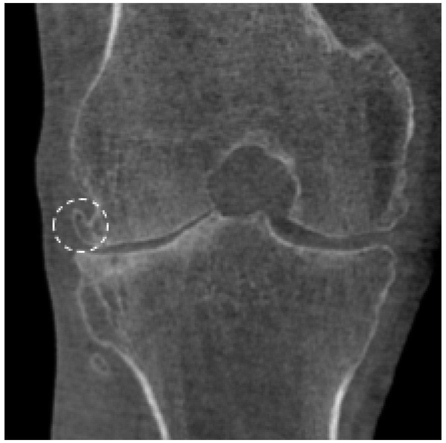	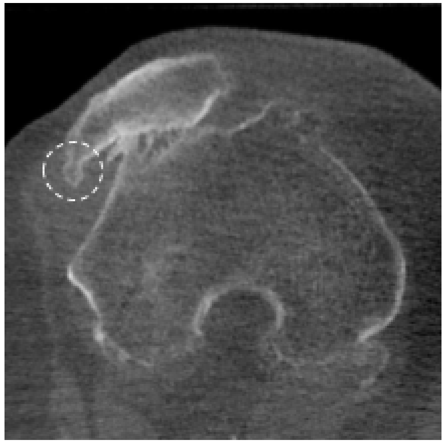	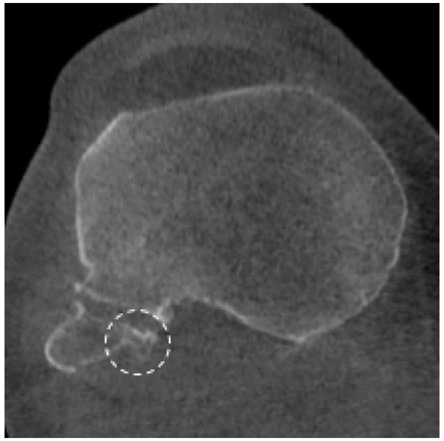

CYSTS

Definition:

"A low-density region in subchondral bone interrupting the normal bony architecture, with or without a sclerotic rim. A connection with the articular surface need not be visible."

Notes:

- Recommended planes for assessment are:
    - coronal and sagittal for tibiofemoral compartments;
    - axial and sagittal for the patellofemoral compartment;
    - axial, sagittal, and coronal for the proximal tibiofibular compartment.
- Cysts of any size in immediate subchondral bone, including the tibial spines, but not deep to the cruciate or meniscal insertions or other sites of intraosseous ganglion formation.
- Be wary of noisy imaging mimicking small cysts.
- Multiple cysts do not have to be present in the same review image for scores 2 and 3, noting that they can be found at multiple locations across the same compartment and not seen in the same image.

CYST GRADE 0 = None			
Medial tibiofemoral (coronal)	Lateral tibiofemoral (coronal)	Patellofemoral (axial)	Proximal tibiofibular (axial)
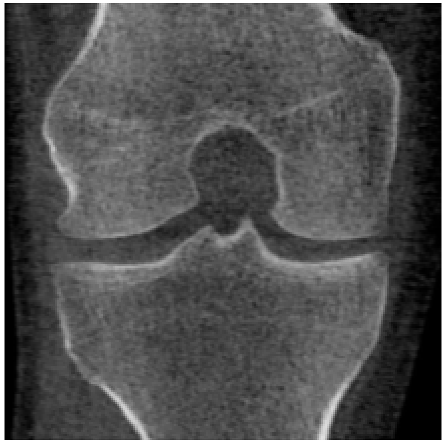	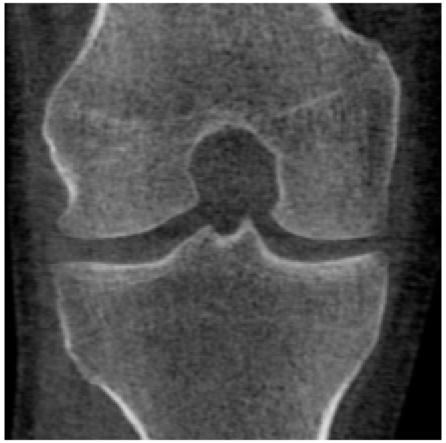	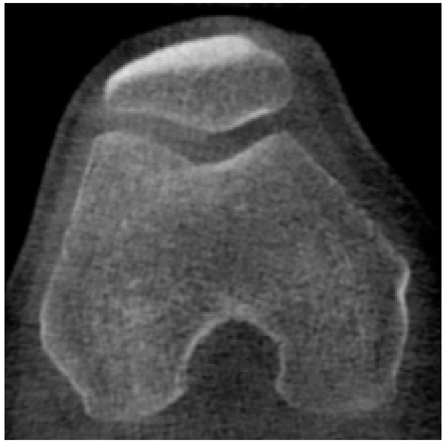	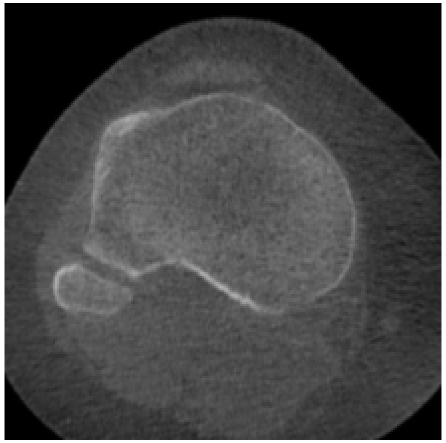

CYST GRADE 1 = One cyst at a single location			
Medial tibiofemoral (coronal)	Lateral tibiofemoral (coronal)	Patellofemoral (sagittal)	Proximal tibiofibular (coronal)
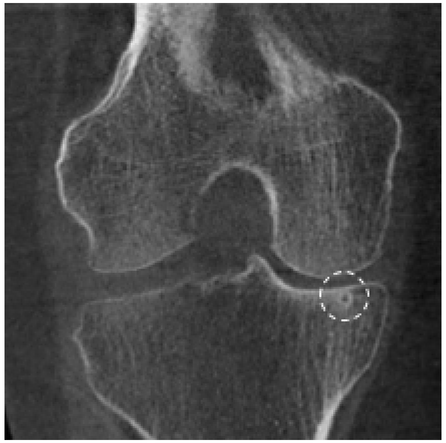	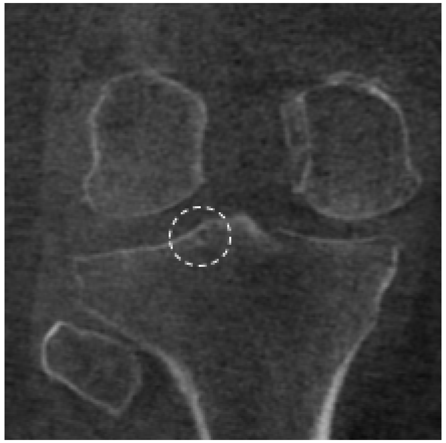	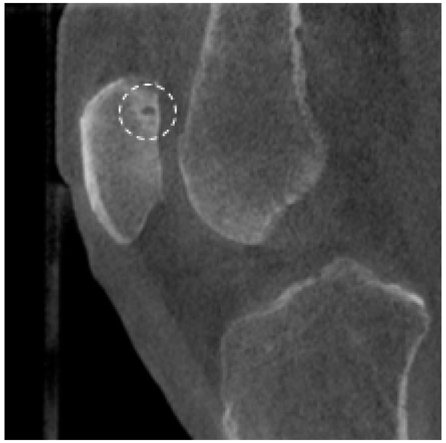	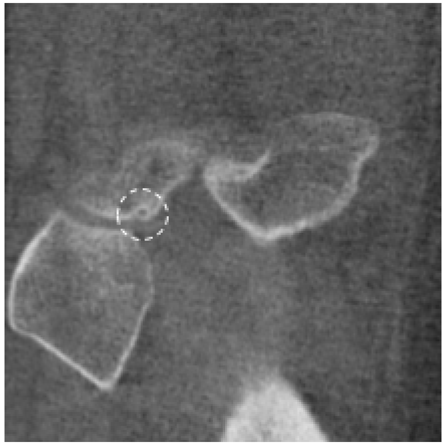

CYST GRADE 2 = Multiple cysts on the same side of the joint			
Medial tibiofemoral (coronal)	Lateral tibiofemoral (coronal)	Patellofemoral (axial)	Proximal tibiofibular (axial)
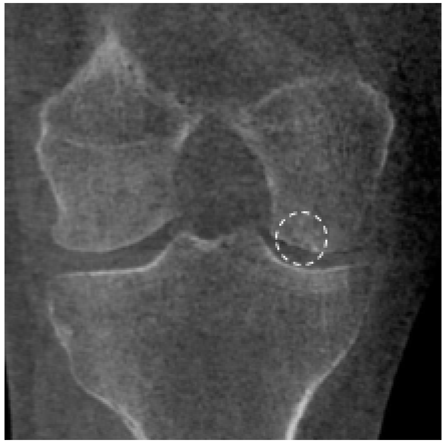	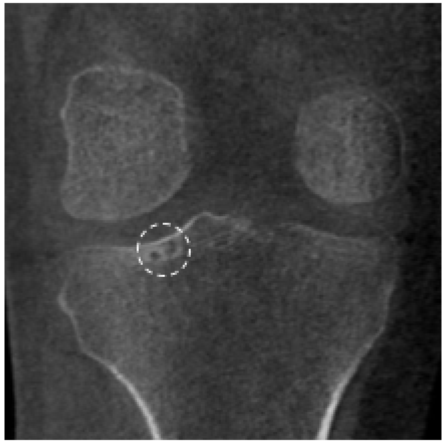	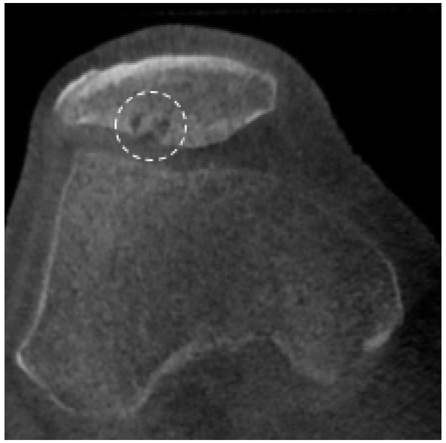	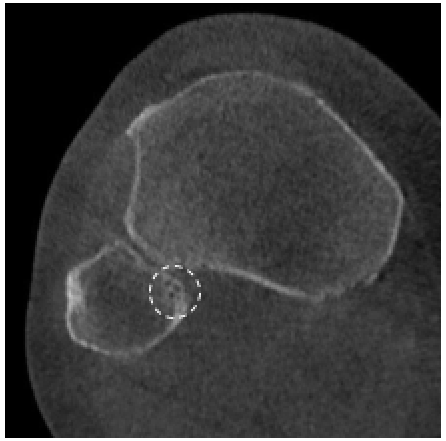

CYST GRADE 3 = Cysts (single or multiple) on both sides of the joint			
Medial tibiofemoral (coronal)	Lateral tibiofemoral (coronal)	Patellofemoral (sagittal)	Proximal tibiofibular (coronal)
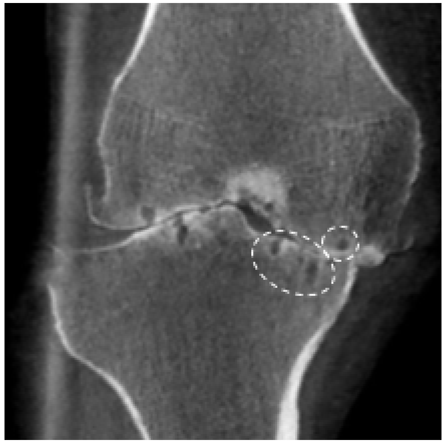	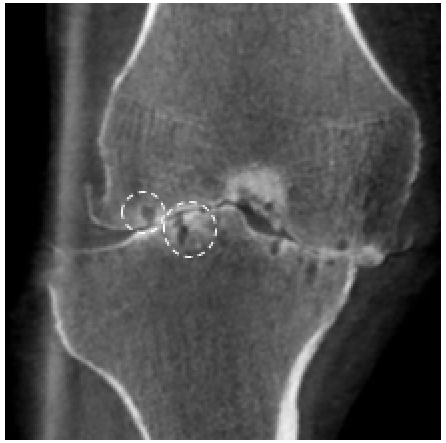	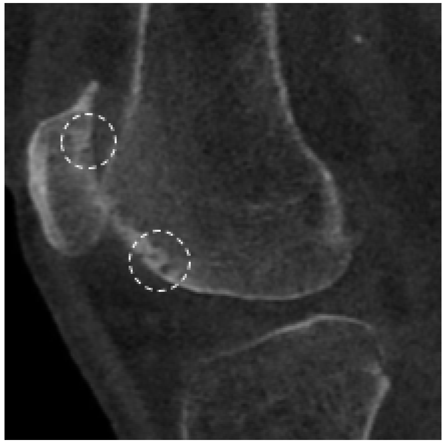	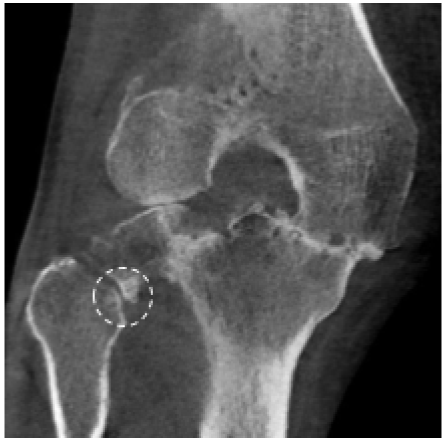

SCLEROSIS

Definition:

"Subchondral bone plate thickening or increased subchondral trabecular bone density beyond normal in load-bearing or contact regions."

Notes:

- Recommended planes for assessment are:
  - coronal and sagittal for tibiofemoral compartments;
  - axial and sagittal for the patellofemoral compartment;
  - axial, sagittal, and coronal for the proximal tibiofibular compartment.
- Be aware of *normal* greater density of subchondral bone through the medial tibiofemoral and medial patellofemoral compartments, which can also appear more pronounced in the context of osteopenia.
- Do not count the sclerotic rim of a cyst, but the surrounding trabecular bone may be scored as sclerotic.

SCLEROSIS GRADE 0 = None			
Medial tibiofemoral (coronal)	Lateral tibiofemoral (coronal)	Patellofemoral (axial)	Proximal tibiofibular (axial)
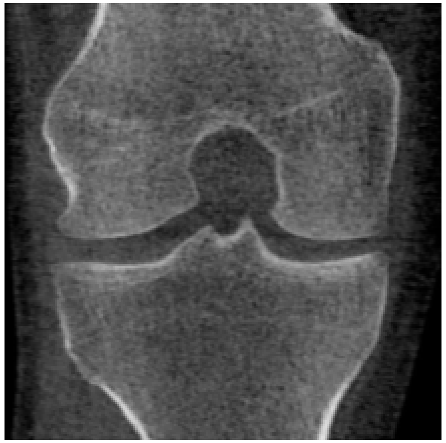	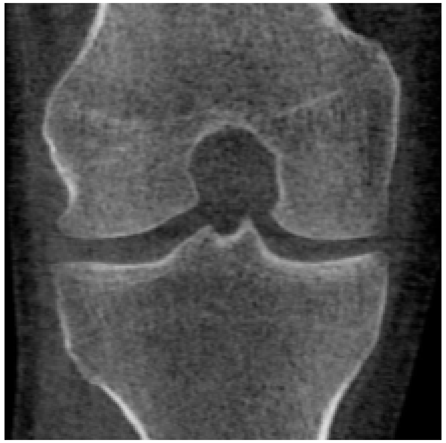	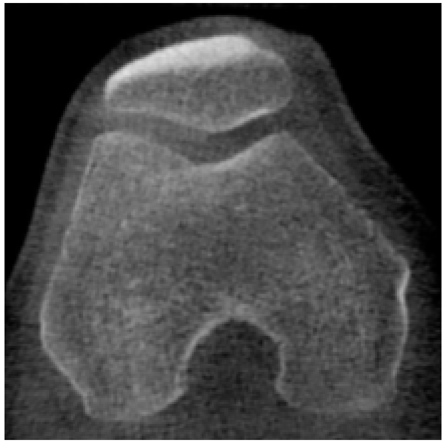	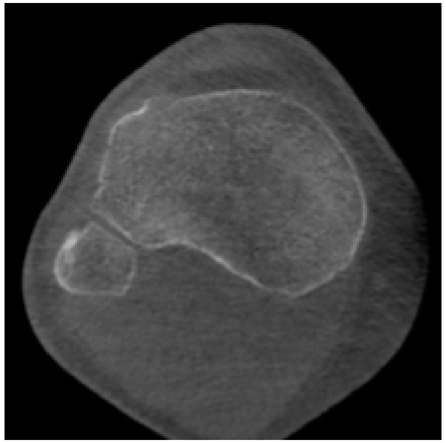

SCLEROSIS GRADE 1 = Possible or suspected			
Medial tibiofemoral (coronal)	Lateral tibiofemoral (coronal)	Patellofemoral (axial)	Proximal tibiofibular (axial)
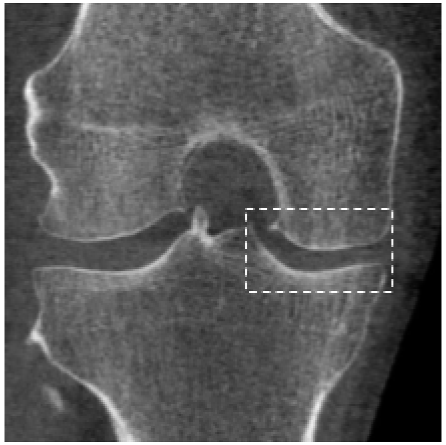	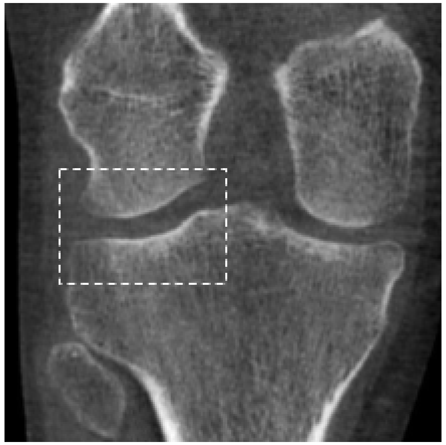	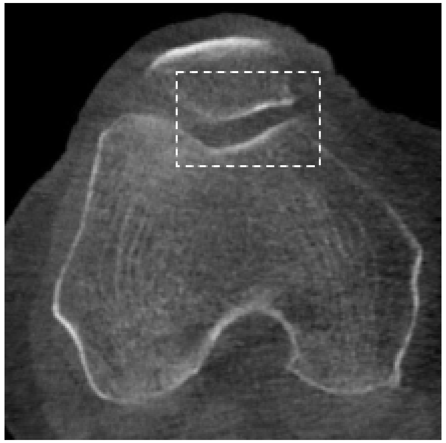	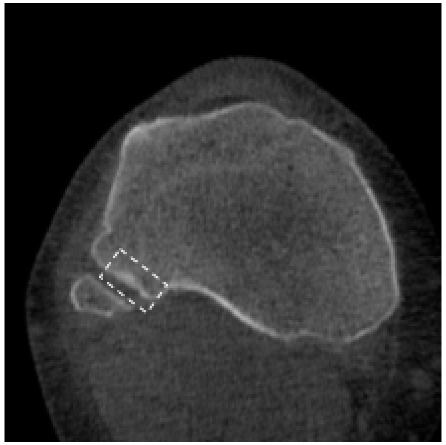

SCLEROSIS GRADE 2 = Definite (but not grade 3)			
Medial tibiofemoral (coronal)	Lateral tibiofemoral (coronal)	Patellofemoral (axial)	Proximal tibiofibular (axial)
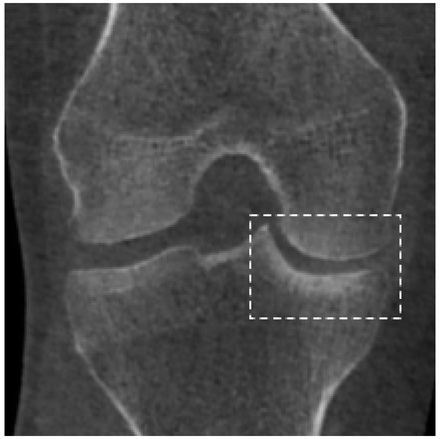	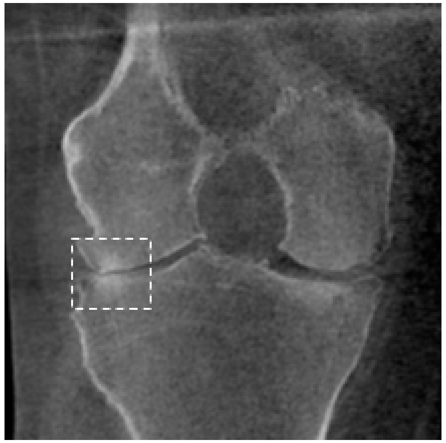	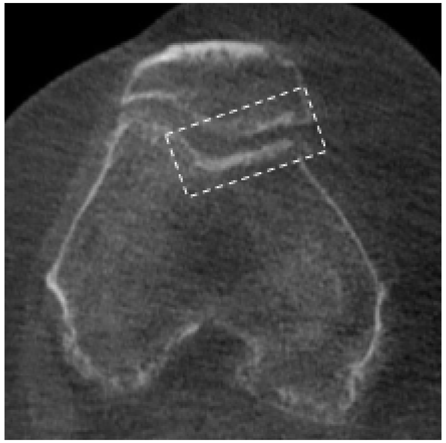	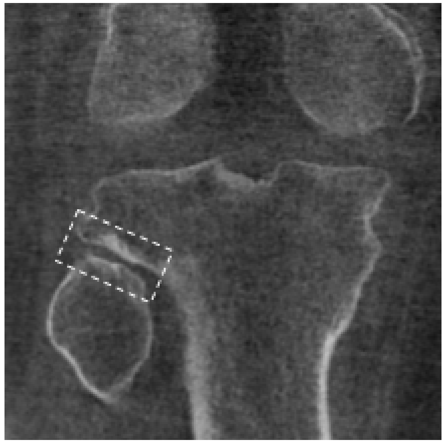

SCLEROSIS GRADE 3 = Severe with bony eburnation, collapse, erosion, or deformity			
Medial tibiofemoral (coronal)	Lateral tibiofemoral (coronal)	Patellofemoral (axial)	Proximal tibiofibular (axial)
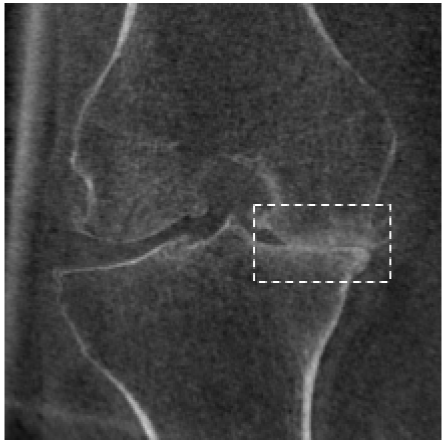	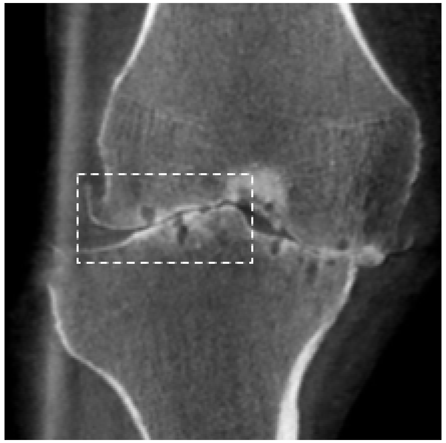	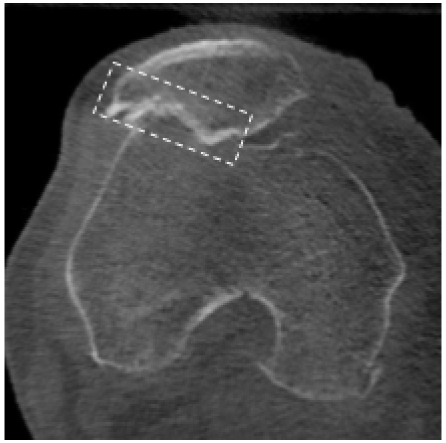	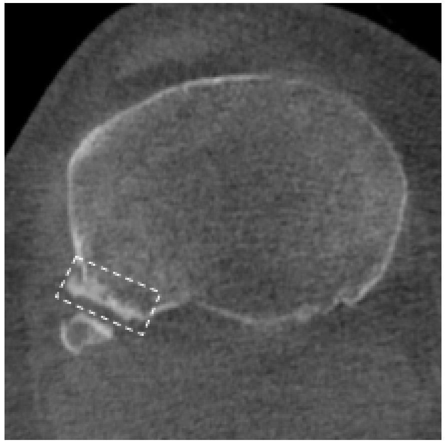

PITFALLS			
Central osteophyte (sag.)	Central osteophyte (ax.)	Enostosis	Fabella (not osteophyte)
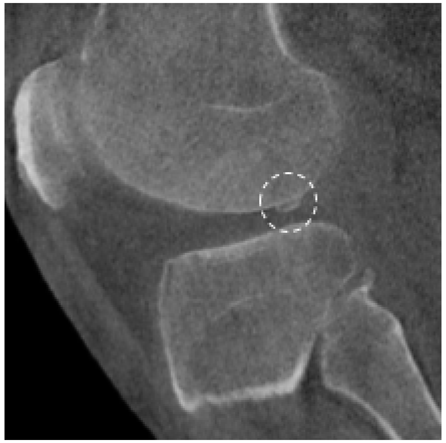	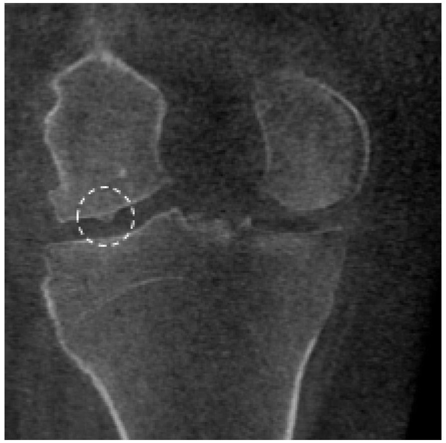	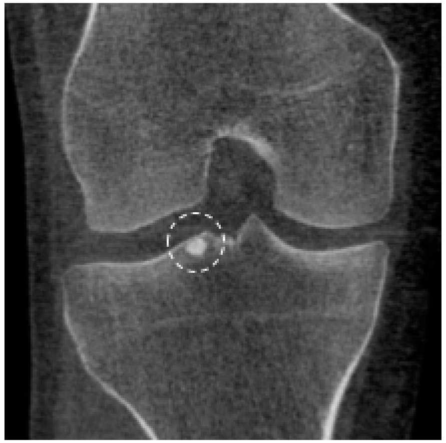	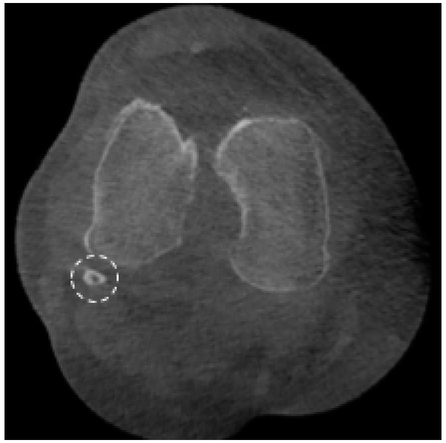
ACL ganglion cyst	PCL ganglion cyst		Suspected subchondral fracture
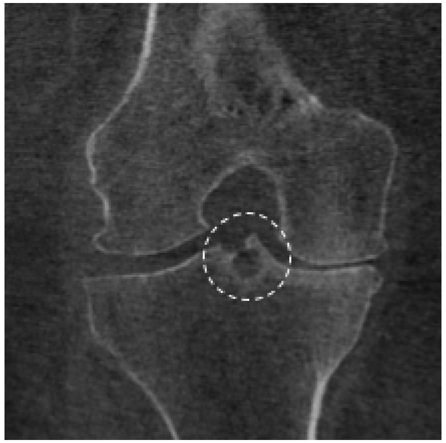	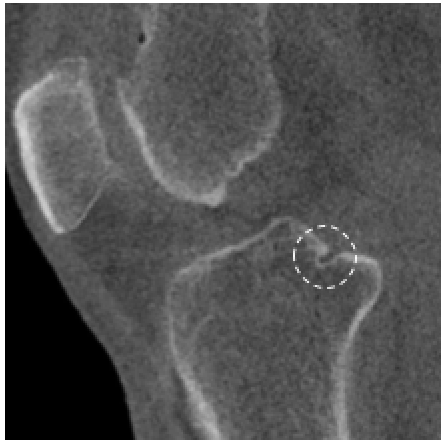		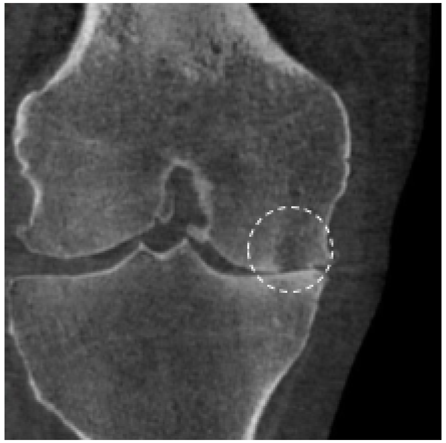

Medial meniscal root cyst (post.)	Medial meniscal root cyst (ant.)	Lateral meniscal root cyst (ant.)	Lateral meniscal root cyst (post.)
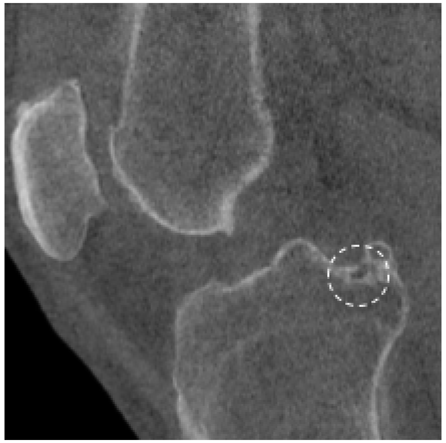	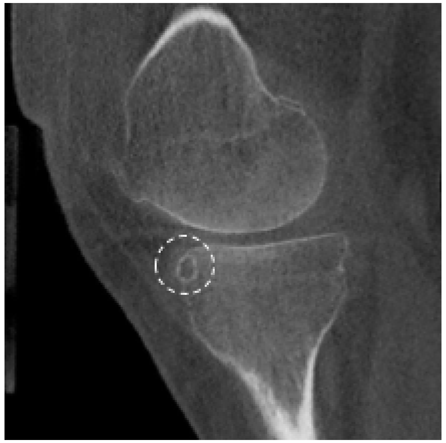	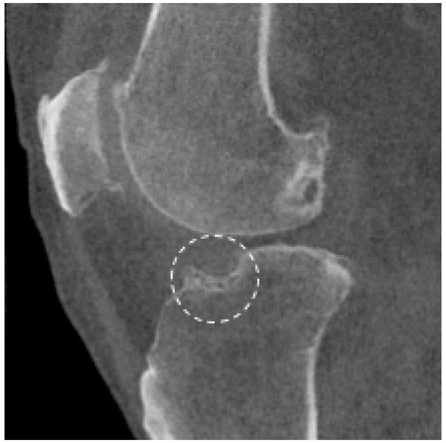	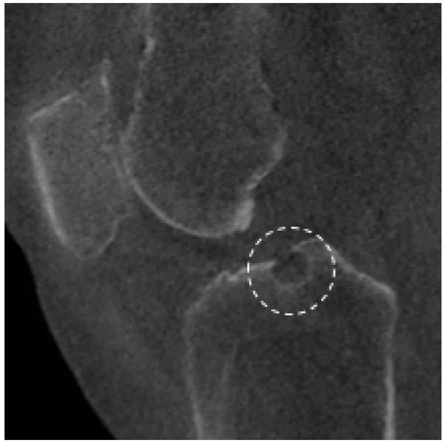
Perforating vessels (femur sag.)	Perforating vessels (femur ax.)	Perforating vessels (tibia)	
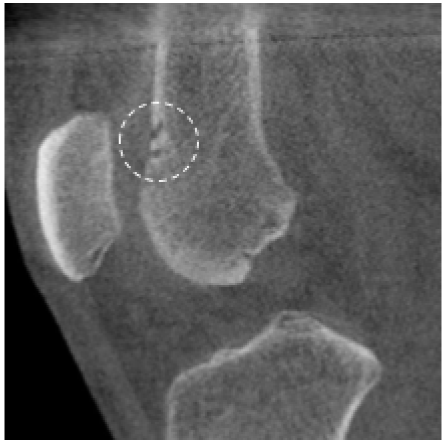	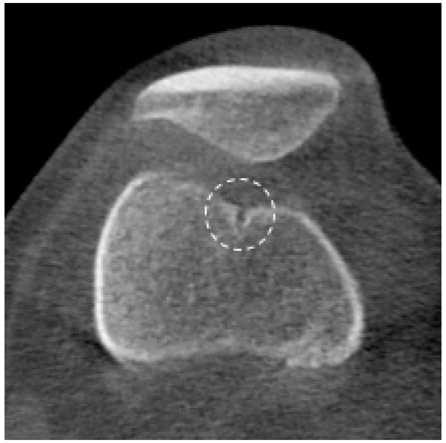	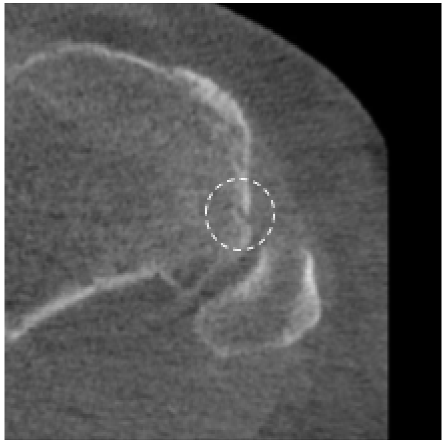	
